# A Step Closer to Meeting the Threat of Avian Influenza

**DOI:** 10.1371/journal.pmed.0030375

**Published:** 2006-09-12

**Authors:** Stacey Schultz-Cherry, Jonathan A McCullers

## Abstract

Schultz-Cherry discusses a new study that describes the generation of live, attenuated H5N1 influenza viruses that may be suitable candidates for use in humans.

In recent years, highly pathogenic avian influenza A viruses of the H5N1 subtype have crossed the species barrier and infected humans in many parts of the world. These strains continue to evolve and expand their host range, and have been associated with greater than 50 percent mortality [1]. No vaccines directed against H5N1 strains are commercially available for humans, and the development of effective H5N1 vaccines poses a number of challenges on both the basic science and manufacturing levels [2].

Several strategies have been explored to produce effective H5 vaccines. These include: preparation of an inactivated vaccine using a heterotypic (see Glossary), low-pathogenicity avian influenza virus that is antigenically related to the H5N1 circulating strains [3]; use of a recombinant H5 hemagglutinin (HA) expressed in baculovirus [4]; an inactivated subvirion vaccine [5]; or the production of attenuated seed viruses with the H5 HA modified through reverse genetics [6–8]. Unfortunately, many of these approaches have been found to be poorly immunogenic, require high doses of antigen, or require the use of an adjuvant [1,9]. An alternative approach under development is the use of live attenuated cold-adapted H5 vaccines.

Live attenuated cold-adapted vaccines are attractive for the prevention of pandemic influenza due to the stimulation of an immune response following a single dose of vaccine, an excellent safety profile, induction of cross-reactive immune responses, and efficacy in children [10]. Although the cold-adapted virus is live, it does not replicate to high titers and fails to readily infect naïve people [11]. Through the use of reverse genetics, a cold-adapted H9 influenza virus–specific seed virus has already been produced and is available for clinical evaluation and use should the need arise [12]. A new study in *PLoS Medicine* now describes the generation of live, attenuated H5N1 influenza viruses that may be suitable candidates for use in humans [13].

## The New Study

In the current work, the authors used reverse genetics to construct high-yield 6:2 seed viruses by mixing the six internal genes from a cold-adapted *(ca)* donor virus with the H5 HA and N1 neuraminidase (NA) from representative H5N1 influenza strains. An important safety feature used in these studies was the modification of the multi-basic amino acid motif at the cleavage site of the H5 HA gene to a sequence seen in avian influenza viruses that are not highly pathogenic in chickens [6]. This alteration affords several advantages, including attenuated growth in embryonated eggs, allowing high yields to be attained, and increased safety due to restricted replication of the virus in the host [2]. The H5N1 ca viruses were attenuated in vivo in mice, ferrets, and chickens as compared to the H5N1 wild-type *(wt)* viruses. The level of replication of the H5N1 *ca* viruses was decreased in the respiratory tract of mice and ferrets as compared to *wt* viruses, and systemic spread was abrogated. The poor infectivity of the H5N1 *ca* viruses in chickens suggests that, if used during a pandemic, the H5N1 *ca* viruses would pose no risk to the poultry industry, an important economic and public health consideration.

Development of effective H5N1 vaccines poses challenges on both the basic science and manufacturing levels.

To evaluate the immunogenicity of the H5N1 *ca* vaccine candidates, each of the viruses was administered to mice and ferrets, and antibody responses were monitored. A single dose of vaccine was poorly immunogenic in mice, generating low antibody responses. However, it fully protected from lethal challenge with homologous H5N1 *wt* viruses, lowered viral titers in the respiratory tract, and prevented systemic spread of the *wt* viruses to the brain. Most excitingly, this single dose provided complete protection from lethal challenge with H5N1 viruses from representative viruses from diverse clades, including the newly isolated 2005 viruses. Administration of a second, boosting dose of the vaccine elicited higher antibody titers with greater cross-reactive responses and afforded near complete protection against not only mortality but viral replication of both homologous and heterologous strains. Two doses of the H5N1 *ca* vaccine candidates also completely protected ferrets from viral replication in the lungs following homologous or heterologous challenge, including challenge with 10^7^ 50% tissue culture infectious dose (TCID_50_) of A/Indonesia/5/2005 (H5N1).

## Importance of the Study

Live, attenuated influenza virus vaccines have been approved for use in healthy persons five to 49 years of age and have been shown to be highly efficacious against influenza-associated lower respiratory tract disease and otitis media in children [11]. They also have significant advantages over inactivated vaccines for immunizing naïve populations, including induction of higher and more durable levels of antibody and generation of cellular and mucosal immunity. This combined humoral, mucosal, and cellular immune response may result in broad protection against antigenically drifted strains, an important advantage since we do not know what future strain will become established in humans and cause the next pandemic. Because prior immunity to the HA of strains contained in the vaccine may limit viral replication and therefore induction of immunity, this approach may actually work better in persons naïve to the virus as is required by definition for a vaccine strain to achieve pandemic status.

The studies by Suguitan et al. provide clinicians with a powerful tool in the fight against pandemic H5N1 influenza viruses: an “off-the-shelf” seed virus that could be standardized, rapidly produced, and safely handled by vaccine manufacturers for vaccine production against a diverse population of H5N1 viruses. The production of H5N1 live attenuated viruses with modifications to the multi-basic cleavage site also provide assurance that these viruses, if shed into the environment, would pose limited risk to the poultry industry and potentially to public health. Although a single dose of vaccine did not induce complete protection from viral replication in the animal model, in the clinical setting this partial protection may translate to protection from severe illness and death in humans.

**Glossary**

**Baculovirus:** A particular virus infecting insects or insect cells. Commonly used for production of recombinant proteins.

**Cold-adapted vaccine:** A virus modified to replicate efficiently only at lower temperatures, resulting in attenuated growth limited to the upper respiratory tract.

**Heterotypic:** Of a different type or form.

**High-yield 6:2 seed virus:** A viral stock containing six viral genes from one strain and two viral genes (typically HA and NA) from the strain of interest.

**Reverse genetics:** Cutting-edge molecular technology used to create influenza viruses from plasmids.

**Seed virus:** A virus used as a “stock” for vaccine production.

## Implications and Limitations of the Approach

In the event of an H5N1 pandemic, will we be prepared? That question looms on the minds of clinicians, researchers, and policy makers worldwide. The approach presented here by Suguitan et al. may represent an important advance in generating effective H5N1 vaccines. However, two potential caveats remain.

First, the immunogenicity of live, attenuated viruses bearing an H5 HA must be established in humans. Other vaccine approaches have protected mice against H5N1 viruses but proved poorly immunogenic in humans. However, the efficacy of this vaccine candidate in ferrets is an important indicator of its potential utility in humans. Second, the risk of reassortment of the vaccine virus with a wild-type human influenza virus must be considered. Although live, attenuated influenza viruses do not transmit well, reassortment within a person vaccinated and then coinfected with wild-type influenza virus could result in a hybrid virus fully competent for replication and transmission in humans that contains the antigenically novel H5 HA, inadvertently triggering a pandemic. This concern will limit clinical testing of the vaccine in humans and may restrict use of this vaccine approach to the period after a new pandemic strain has begun to circulate. Nonetheless, the work from Suguitan et al. is an important step along the path to influenza preparedness and warrants further development.

## Abbreviations

*ca*: cold-adapted
HA: hemagglutinin
NA: neuraminidase
wt: wild-type
